# Different Potent Glucocorticoids, Different Routes of Exposure but the Same Result: Iatrogenic Cushing’s Syndrome and Adrenal Insufficiency

**DOI:** 10.4274/jcrpe.galenos.2020.2019.0220

**Published:** 2020-11-25

**Authors:** Ayla Güven

**Affiliations:** 1 University of Health Sciences Turkey, İstanbul Zeynep Kamil Women and Children Diseases Hospital, Clinic of Pediatric Endocrinology, İstanbul, Turkey

**Keywords:** Cushing’s syndrome, adrenal insufficiency, glucocorticoids, adverse effect, hypercalcemia, non-alcoholic fatty liver disease

## Abstract

**Objective:**

Potent glucocorticoids (GC) cause iatrogenic Cushing’s syndrome (ICS) due to suppression of hypothalamo-pituitary-adrenal (HPA) axis and may progress to adrenal insufficiency (AI). The aim was to review the clinical and laboratory findings of patients with ICS and to investigate other serious side effects.

**Methods:**

The possibility of AI was investigated by low-dose adrenocorticotrophic hormone test. Hydrocortisone was started in patients with adrenal failure.

**Results:**

Fourteen patients (five boys) with ages ranging from 0.19 to 11.89 years were included. The duration of GC exposure ranged from 1 to 72 months. Ten patients were prescribed topical GC and the rest had oral exposure. Moon face and abdominal obesity were detected in all patients. At presentation, 12 of 14 had AI and two infants had hypercalcemia and nephrocalcinosis. Of 11 patients, ultrasonography revealed hepatosteatosis in five. A cream for diaper dermatitis was used in one infant and the active ingredient was listed as panthenol. However, blood and urine steroid analyses revealed that all endogenous steroids were suppressed. Median (range) time to normalization of HPA axis function was 60 (30-780) days.

**Conclusion:**

The majority (85%) of patients had life-threatening AI and two patients had hypercalcemia. These results highlight the serious side-effects of inappropriate use of potent GCs, especially in infants. The recovery of the HPA axis in children might take as long as three years. Parents should be informed regarding the possibility of some products containing unlisted synthetic GC and to be aware of their side effects.

**What is already known on this topic?**

The most common cause of Cushing’s syndrome (CS) in childhood is the administration of high doses of synthetic glucocorticoids (GC) for treatment purposes or misuse of these steroids.

**What this study adds?**

This is the largest series presenting iatrogenic CS (ICS) and adrenal insufficiency (AI) caused by potent steroids in childhood. AI is a rare cause of hypercalcemia in infancy and childhood but hypercalcemia was detected in two infants of fourteen patients in this study. In addition, an infant with ICS had exposure to a cream and the patient’s urine and blood steroid analyses revealed exposure to high-dose steroids.

## Introduction

Cushing’s syndrome (CS) is very rare in childhood and the most common cause is the administration of high doses of synthetic glucocorticoids (GC) for treatment purposes or misuse of these steroids (1). GC are one of the most widely used drugs in the treatment of numerous diseases, including hematological diseases, oncological malignancies, respiratory system diseases, rheumatologic diseases, neurological diseases, kidney diseases, organ transplantations and adrenal insufficiency (AI). As the potency and duration of administration of GC increase, the risk of serious side effects also increases. These side effects include hypercortisolism, hypothalamo-pituitary-adrenal axis (HPA) suppression, non-alcoholic fatty liver disease (NAFLD), osteoporosis, or even adrenal atrophy. Hypercortisolism due to exogenous steroids is known as iatrogenic CS (ICS) (2).

Synthetic GC exogenously given via oral, iv, intramuscular, intra-articular, topical, inhaled, intra-ocular or intra-nasal routes can cause ICS. It is known that GC, which are frequently administered orally and topically in childhood, cause ICS (3,4). In particular, application of topical steroids to the diaper region of an infant may lead to CS and subsequent AI (5). Such use of exogenous GC may even facilitate the spread of infectious disease through suppression of immune function that might be fatal (6). In addition to potent steroids, long-term and high-dose administration of low-potency GC such as hydrocortisone (HC), prednisolone (PZ) and methylprednisolone (MPZ), which are frequently used in the treatment of several diseases, may cause similar side effects (7,8).

In this article, the clinical and laboratory findings of 14 patients with ICS due to oral GC treatment prescribed by physicians for treatment purposes and topical steroids applied by their parents are presented. The aim of this study was to review the clinical and laboratory findings of patients with ICS and to investigate and demonstrate other rare but important side effects.

## Methods

### Patients

In this retrospective study, all data was obtained from patients’ medical records. Only those exposed to high-dose potent GC were included in this study. Anthropometric measurements were recorded. Body mass index (BMI) of the patients was calculated. Height, weight and BMI-standard deviation (SD) score (SDS) of the patients were calculated using “Child metrics” (9). Fourteen patients including nine girls and five boys, aged between 0.19 and 11.89 years, were identified. All patients had been given a high dose of moderate to high potency GC either orally or topically. Those who were exposed to topical potent GC for more than a week or had used oral potent steroids for more than 15 days were included in the study.

Ten patients had been given topical GC, such as clobetasol-propionate, diflucortolone-valerate, MPZ-aceponate and betamethasone exposure. The remaining four had been given MPZ and PZ orally.

The mother of one patient (case 12) had been using an ointment to prevent diaper dermatitis since birth, believing that it contains panthenol. The manufacturer stated that there was no GC in the cream.

In addition to the side effects of steroids in these patients, AI due to suppression of the HPA axis, frequently seen in ICS patients, was investigated. Low-dose adrenocorticotrophic hormone (LD-ACTH) test was performed to investigate AI in all patients except two. These two were not tested because the family of one did not consent and the other patient, with severe thrombocytopenia, could not be tested since intravenous (iv) Synacthen was not available at the time.

The equivalent daily dose (EDD) of GC exposure was calculated in five patients, according to HC equivalence. However, the EDD could not be predicted for those who were exposed to topical steroids. The potency of GCs according to HC was determined (2).

Ophthalmologic examination was performed in seven patients and abdominal ultrasonography was performed in 12 patients. Detailed clinical information about all patients is given in supplemental file.

### Laboratory Investigations

After an overnight fast (in children for at least eight hours and in infants for at least six hours) blood samples were taken. Serum concentrations of glucose, calcium, phosphorus, aspartate transaminase (AST), alanine transaminase (ALT), alkaline phosphatase, total cholesterol (TC), low-density lipoprotein (LDL-C), high-density lipoprotein (HDL-C) and triglyceride (TG) were measured. Fasting concentrations of serum TC, TG and LDL-C were considered high when these were above 200 mg/dL, 100 mg/dL and 130 mg/dL, respectively. The desirable level of HDL-C was above 40 mg/dL, so the cut-off point was accepted as 40 mg/dL. Serum parathyroid hormone, magnesium, 25-hydroxyvitamin D [25-(OH)D3] and 1,25-dihydroxyvitamin D [1,25-(OH)_2_D_3_] levels were measured in the two patients with hypercalcemia. Spot urine calcium and creatinine ratio also was calculated.

A 24-hour urine sample and a morning fasting blood sample were obtained from case 12 before treatment was initiated. Blood steroid analysis was performed at Marmara University Medical Faculty Hospital, Biochemistry Laboratory and high-performance liquid chromatography was used in the analysis. In addition, urine steroid metabolites of this infant were measured using quantitative gas chromatography-mass spectrometry in selected ion monitoring at the University of Birmingham, College of Medical and Dental Sciences, Steroid Metabolome Analysis Core, Institute of Metabolism and System Research.

A LD (1 µg) ACTH test was performed in 12 patients (Synacthen 250 µg, iv, Novartis, Basel, Switzerland) for adrenal investigation. A stimulated peak cortisol level on LD-ACTH test of more than 500 nmol/L (equivalent 18 µgr/dL) was considered adequate (10). Lower levels of cortisol (<500 nmol/L) demonstrate AI. In patients who received HC, MPZ or PR with a drug reduction scheme, the LD-ACTH test was performed 4-7 days after the discontinuation of the drug.

### Therapy and Follow-up

Nine of the patients had been treated with HC (15-20 mg/m^2^/day), and one patient with PR (4-5 mg/m^2^/day) for 2-3 weeks for prevention of GC withdrawal syndrome. Case 1 had been treated with MPZ (15 mg/m^2^/d), since a total of 5000 mg MPZ po was given over five days for severe immune thrombocytopenic purpura before admission. HC, MPZ or PR doses were gradually decreased over the 2-3 weeks. According to the age of the patients, LD-ACTH test was re-performed with an interval of one to two months. The HPA axis was considered to be recovered when stimulated cortisol increased above 18 µgr/dL.

Written informed consent was obtained from the parents. The hospital ethics committee approved this study (Zeynep Kamil Women and Children Diseases Training and Research Hospital Clinical Research Ethics Committee, 116/18.12.2019).

### Statistical Analysis

All analysis was done using the Statistical Package for the Social Sciences, version 21 (IBM Inc., Armonk, NY, USA). In order to determine whether the data was normally distributed the Shapiro-Wilk method was used. Descriptive statistics of the data which were normally distributed are summarized as mean±SD. For non-parametric data summary is given as median (interquartile range: IQR). For all tests, a p-value of less than 0.05 was accepted as statistically significant.

## Results

A clinical summary of the patients is given in Table 1. The median age was 1.76 (11.05) years (0.19-11.89). Mean BMI of the patients was 24.3±7.8 kg/m^2^, median height was 79.7 (80.23) cm and median weight was 12 (58.5) kg. Mean height SDS of the patients was -0.46±1.32, weight SDS was 1.32±2.1, and BMI SDS was 1.82±2.0. Only one patient had short stature, a girl who received high dose MPZ orally for two years; all other patients had height or length within the expected range. At presentation, six patients (four girls) were obese and three were overweight. However, BMI SDS of the two infants were -1.44 and -1.63. Systolic and diastolic blood pressure was normal in all patients in whom blood pressure could be measured (mean systolic: 108±15 mmHg and mean diastolic: 69±4.9 mmHg).

Five patients were exposed to potent GCs orally, and nine patients via the transdermal route. Median duration of high dose GC exposure was 4 (22.1) (range 1-72) months. In five patients who were exposed orally the equivalent HC daily dose was 93±78 mg/m^2^/d. However, the EDD could not be estimated in nine patients, since the amount applied to skin and its absorption could not be calculated.

Median basal ACTH and cortisol levels of the patients were 16.9 (22.91) pg/mL and 3.73 (6.37) mcg/dL, respectively. Mean stimulated cortisol was 8.55±6.5 mcg/dL. At presentation, 100% of the infants were found to have AI, while overall 85% of patients had AI.

Persistent AI was detected in case 2 due to adrenal atrophy. On presentation the HPA axis was normal in two patients. However, HPA axis normalization time was not predicted in three patients because they were lost to follow up after initial discharge. In the remainder the normalization of the suppressed HPA axis occurred at a median of 60 (IQR 160; range 30-780) days in nine patients. Patients were followed for a median of 240 (825) days.

Although the majority of the patients’ transaminases were within normal range (AST 31.1±14 IU and ALT 35.3±23 IU), these were elevated in two patients. Mean fasting serum TG level was 131±63 mg/dL. Hypertriglyceridemia was detected in six of nine patients whose fasting TG levels were measured. The youngest patient with hypertriglyceridemia was two months old. Hypercholesterolemia and increased LDL-C was found in the same two patients. Low HDL-C was detected in other two patients (Table 2). AI was detected in 12 patients with serum basal and/or stimulated cortisol (Table 2). Bilateral posterior segment cataract was detected in case 1 on ophthalmologic examination. Abdominal ultrasonographic examination was performed in 11 patients. Five patients had hepatosteatosis. Two patients with hypercalcemia had nephrocalcinosis. In case 12, all adrenal steroids including androgen precursors and GC and their precursors were found to be decreased in the blood steroid profile. The urine results also showed low androgen precursors, GC and their precursors. TH-aldosterone was slightly increased above normal, but others were normal or low. This profile was not consistent with endogenous CS, but compatible with exogenous administration of GC and subsequent suppression of endogenous GC production.

## Discussion

In this study, clinical and laboratory results of children who were prescribed high-dose and potent GC by a doctor or given them by their parents were investigated. In the present study, six of the 14 patients were younger than two years of age and five of them were exposed to high-dose GC via the transdermal route. Topical steroids were administered to these patients by their parents to prevent or treat diaper dermatitis. An LD-ACTH test was used in all of them except for case 5, and all revealed AI. In infants diagnosed with AI, low potency GC, such as PZ and MPZ, as well as high potency GCs, such as diflucortolone valerate 0.3% and clobetasol propionate 0.05% were used.

It is accepted that GCs, when used traditionally in doses less than 20 mg/day for less than three weeks, will not cause AI. However, it was reported that AI might be seen in adult patients who are exposed to prednisone or an equivalent dose of GCs at doses greater than 20-30 mg/day for more than five days (11). In addition, studies in adults have shown that high-dose GC therapy can cause HPA axis suppression even with a five day-treatment (12). Different results have been published regarding the recovery times from HPA axis suppression in children. In children with asthma who received PZ for five days, a full improvement in HPA axis was observed in 10 days, whereas in infants who received 12-25 weeks of high dose PZ, this period was 6-12 weeks (13,14). Moreover, it has been demonstrated that the HPA axis returns to normal within 1-2 weeks following discontinuation of PZ treatment for more than six months (15). In this study, it was found that the HPA axis returned to normal between 30 and 780 days.

AI might also occur in patients exposed to topical, inhaled, or intra-nasal GCs, depending on dose and potency (3,4). Patients in this series had been exposed to potent GCs for at least seven months. AI was inevitable due to the patients being children, and half of them were infants, and the vast majority of them were exposed to topical potent-GCs with unpredictable doseage, due to the route of application.

Topical GCs with mild and moderate potency, used in children to treat atopic dermatitis, have rarely been demonstrated to suppress the HPA axis, even if used for a long time (16,17). However, potent or high-potency topical GCs or combinations of other GCs are well recognized to suppress the HPA axis and cause AI (3,4). In addition, it has been shown that the severity of HPA suppression was negatively correlated with cortisol response in the LD-ACTH test (17). Suppression of the HPA axis is dose dependent after systemic GC treatment (18). In older children, potent GCs usually cause mild to moderate AI, to a greater degree than HC would, but they rarely cause severe AI (16).

Although obesity is an expected finding in patients with CS, seven patients had a BMI SDS <+2 in this study, the three infants and one child . Two of these infants were underweight for their age. The reasons for not gaining weight, such as malabsorption, food intolerance, or gastroesophageal reflux in these patients could not be investigated. The reason for weight loss may be central AI caused by suppression of the HPA axis by potent GCs. Anorexia, nausea, and weight loss are well-known signs of AI (19,20) and may be the reason for poor weight gain in these infants. In addition, the girl infant was born prematurely, was followed up in the neonatal intensive care unit and had a history of cardiac arrest on the 4^th^ day of her life. She was being followed up with a diagnosis of ichthyosis since birth. On admission, she was still being treated with a number of potent GCs simultaneously by the dermatologist. At presentation there was no sign of catch-up growth. These findings do not permit comment on the underlying reasons for failure to gain weight in infants in this study.

In this study, AI was absent on admission in two patients who were exposed to potent steroids. Clobetazol-containing pomade was not applied to case 13 for two months prior to presentation. Although she was obese, the cause of the absence of AI on LD-ACTH test was the improvement in the HPA axis within these two months. Oral MPZ was given to case 14 for treatment of asthma for a month. A month before his admission, MPZ treatment was abruptly stopped due to weight gain. In this patient, AI was not detected at admission since the duration of MPZ usage was short and it is possible that HPA axis function improved within this one month respite from MPZ therapy. Depending on the dosage of GCs, the route of administration, and the duration of drug administration, complete recovery of the HPA axis after discontinuation may vary from one week to several weeks (12,13,14,15). We hypothesize that the reason that AI was not detected following ICS in these patients was the complete recovery of HPA in the period between discontinuation of GC and their admission to the clinic.

The metyrapone test is not recommended in the diagnosis of AI in children, and is even more strongly discouraged in infants, since it could trigger adrenal crisis. Furthermore, the insulin tolerance test (ITT) is inconvenient in younger children, and in patients with a history of seizure or cardiac insufficiency (21). Since both the ITT and the metyrapone test carry major risks, such as precipating acute AI, corticotrophin analog stimulation test has been introduced. The LD (1 µg of corticotrophin) ACTH stimulation test is easier to perform than the ITT and carries a very low risk of side effects (10,22). In a recent study in adults, the stimulated cortisol values of the 1 mcg ACTH test were compared and it was suggested that the number of false positive patients would be significantly reduced by accepting the stimulated cortisol cut-off value of 401.5 nmol/L (14.55 mcg/dL) instead of 500 nmol/L (18.12 mcg/dL) (23). However, in studies conducted in children, it was stated that values above 500 nmol/L (8) or 550 nmol/L (24) of stimulated cortisol in the 1 mcg ACTH test may exclude AI. In this study, in 10 of the 12 patients tested, the stimulated cortisol response was inadequate, whereas in only two patients, stimulated cortisol was greater than 18 mcg/dL.

It has been shown that GCs increase lipid production in rats and also increase circulating TG, as well as resulting in hepatosteatosis (25). GCs increase the conversion of carbohydrates to fatty acids in hepatocytes, and decrease fatty acid oxidation and also cause an increase in the synthesis of TG in hepatocytes using increased fatty acids (26). In the liver of a baby with AI caused by topical clobetasol propionate, we demonstrated for the first time that macrovesicular fat was present in the liver (6). After our original report, a subsequent study regarding non-alcoholic hepatosteatosis (NAFLD) detected by ultrasonography in infants with ICS was published (5). In the present series, NAFLD was detected in six of 11 patients who had ultrasonographic examination. The youngest patient with NAFLD was 2.2 months old, while the others were older than nine years. The mother of the baby with NAFLD was applying a cream to prevent diaper dermatitis. Blood and urine samples taken before treatment revealed that the synthesis of this patient’s endogenous steroids was suppressed. Since this patient had not been prescribed any GC treatment, it was suggested that the cream used for diaper dermatitis contained potent GC. Misuse of a potent corticosteroid was responsible for hepatosteatosis in our patients. Hypertriglyceridemia, as well as an increase in transaminases, are expected findings in patients with NAFLD (26,27). In the present study, increased transaminase associated with hypertriglyceridemia level was detected in an infant without NAFLD. Interestingly, serum lipids and transaminases were normal in another infant with NAFLD. Among the patients presented here, hypertriglyceridemia was detected in two thirds of the patients in whom this was measured. These abnormal lipid profiles might be due to exposure to potent GCs.

Hypercalcemia has been reported in patients with primary or secondary AI (28,29,30). In addition, the reduction of glomerular filtration due to fluid loss in adrenal crisis, acute kidney injury and hypercalcemia were also found in adult patients (31,32,33). Decreased glomerular filtration results in a reduced filtered load of calcium, and increased calcium renal reabsorption occurs, due to volume depletion in AI. Enhanced calcium mobilization from bone in AI also contributes to the development of hypercalcemia. The postulated mechanism for AI to cause hypercalcemia is through a combination of increased calcium flux into the extracellular space and reduced calcium renal excretion (34). Stanniocalcin, secreted from the adrenal gland, reduces circulating calcium (35). The level of calcium is also reduced due to a decreased production in stanniocalcin in the state of AI. Endogenous GCs decrease the absorption of calcium from the intestines and increase the excretion of calcium in the urine (36). It is postulated that increased calcium absorption is caused by GC insufficiency (37) and GC replacement therapy has been shown to improve hypercalcemia.

In the presented series, hypercalcemia was detected in two infants. Both patients were less than three months old and had been exposed to GCs since the first days of their lives. Vitamin D hypervitaminosis and subcutaneous fat necrosis were not detected in these patients. Nephrocalcinosis was found in both babies. Nephrocalcinosis persisted in one patient until the age of 2.5 years when he was lost to follow-up. CYP24A1 mutation was investigated in this patient and no mutation was detected. The other baby’s father also had nephrolithiasis. However, nephrocalcinosis resolved by 30 months of age in this second case. AI due to exposure to exogenous potent GCs may be the cause of nephrolithiasis in these infants.

### Study Limitations

The first limitation was that not all patients were examined for possible side effects of GC, such as intracranial benign hypertension, osteoporosis, myopathy, glaucoma and neuropsychiatric symptoms (depression, mood changes). The second limitation was that not all cases could be followed up for a long time.

## Conclusion

This study highlights, yet again, the high frequency of AI in children exposed to high-dose oral and topical potent GCs. Recovery of HPA axis function from this AI may be as long as 780 days in children. It is notable that NAFLD was detected even in very young infants exposed to potent GC. It should be also kept in mind that hypercalcemia and nephrocalcinosis may be detected following exposure to potent GC. Clinicians should alert parents to the potentially serious side effects, when prescribing GC to their children.

## Figures and Tables

**Table 1:**
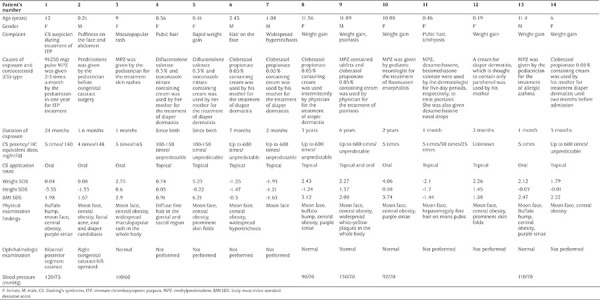
Clinical findings of the patients with iatrogenic Cushing’s syndrome at presentation

**Table 2:**
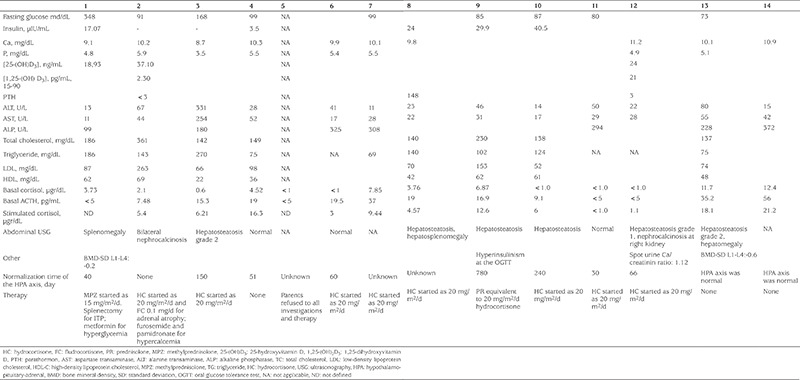
Laboratory findings in the patients
